# Examining a Holistic Framework for Evaluating Clinical Outcomes in Parallel With Non‐Clinical Outcomes

**DOI:** 10.1111/jep.70047

**Published:** 2025-03-06

**Authors:** Robert G. Lingard, Louise Horstmanshof

**Affiliations:** ^1^ Faculty of Health Southern Cross University Lismore New South Wales Australia

**Keywords:** Common Good, Economies of Worth, evaluations, framework, interventions, outcomes

## Abstract

**Rationale:**

While clinical research seeks to evaluate outcomes of various types, no framework has been identified that permits a sufficiently broad approach to evaluating clinical outcomes, in parallel with non‐clinical outcomes.

**Aims and Objectives:**

The objective of this paper is to examine a unifying framework for evaluating clinical outcomes in parallel with non‐clinical outcomes by drawing on different understandings of the Common Good. The proposed framework must have broad application, accounting for the various types of outcomes that may emerge within different disciplinary approaches and identifying the benefits or harms that might be experienced by the clinical research participants.

**Method:**

Six different definitions of the Common Good originally described by Boltanski and Thévenot are presented. The six conceptions of the Common Good identify organising principles by which an outcome is evaluated as beneficial or harmful. It also identifies the various ways that the researched persons, and the researchers, are subjectified. Academic literature that reported outcomes for persons living with dementia was purposively sampled to demonstrate the application of the six definitions of the Common Good.

**Results:**

A wide range of outcomes from clinical research may be evaluated in parallel, based upon the assumption that the Common Good may be expressed as a plurality of perceived goods extending beyond purely clinical, technically measured outcomes.

Further, the subjects of interventions may be described in non‐clinical language, thus respecting the many roles that may be important to them as human persons and agents.

**Conclusion:**

Boltanski and Thévenot's Economies of Worth framework allows research outcomes to be evaluated consistently against the six definitions of the Common Good. The definitions of the Common Good inhere six different Worlds: Civic, Domestic, Industry, Inspiration, Market and Opinion (or Celebrity). Each World is structured around a Higher Common Principle by which the Common Good is defined within that specific world.

The example of research amongst people living with dementia demonstrates the robustness of this framework by identifying a wide range of non‐clinical outcomes. These extend the understanding of the practical ways in which participants, their families and associates, researchers, and also organisations, are variously presented as subjects according to the different definitions of the Common Good.

**Implications:**

This theoretical approach has the potential to inform and support research in clinical settings and guide funding bodies in the evaluation of research projects that give rise to clinical and non‐clinical outcomes.

A matrix that applies the structure of the definitions of the Worlds could be developed to support the comparison of research outcomes arising from diverse objectives and methodologies.

## 1 Introduction

This paper identifies the absence of a framework to guide the evaluation of clinical research outcomes alongside non‐clinical outcomes. It proposes a unifying framework to support evaluation between projects and across disciplinary boundaries. For example, a reading of current literature suggests that the outcomes of interventions and research amongst people with dementia and their carers are generally reported within disciplinary constraints. There is no overarching framework that has been applied to outcomes, regardless of their disciplinary embeddedness that accounts for clinical and non‐clinical outcomes. It is assumed that research participants, researchers and others are seeking to contribute to the Common Good, which often means presenting technical measures to justify the intervention. Outcomes are reported and are asserted to be good and beneficial to the participants, to the wider community, or to the research project itself. Alternatively, outcomes might challenge the rightness of previously held positions in order to ensure a good result. However, the assumptions that constitute the Common Good might not be made explicit, thereby preventing the evaluation of the different types of outcomes that might be of value when observed. Where technical, clinical definitions dominate, the human subjects receiving clinical interventions are confined to clinical definitions of who they are as patients. However, when clinical and non‐clinical understandings are simultaneously considered in parallel, recognising the human subject as, for example, both a patient and a grandmother might enrich the interaction and suggest broader options for care. The example of research outcomes amongst people living with dementia, and their carers, is used to illustrate and ground this otherwise theoretical discussion.

Definitions of the Common Good are taken from the theoretical work of social theorists, Boltanski and Thévenot [1], that embeds these within the understandings of what is tacitly presented in common social interactions. The original description of the Economies of Worth (EoW) framework identified six different regimes, or Worlds of Valuing, by which the Common Good may be appealed to in ordinary interactions among agents who are seeking to justify their actions, or to defend their positions. Each World of Valuing is structured around a Higher Common Principle by which justice is defined. The definitions and descriptions of the Worlds of Valuing follow a common grammar, or pattern and structure. The definitions describe the ways in which subjects and objects are arranged in relation to each other. They further identify principles by which subjects are attributed greater or lesser status. They also propose tests by which a legitimate claim to justice may be made, according to the definition of the Common Good specified within a particular World of Valuing. Boltanski and Thévenot [1] assert that there is a plurality of Worlds that exist simultaneously, and is referenced within social settings.

Alternative frameworks have been applied in research with people living with dementia, for example. Low and Purwaningrum [2] analysed the framing of dementia within a range of literature, media and social media to investigate presentations of dementia across a 20‐year time span to 2018. They reported that dementia was generally negatively presented within the ‘biomedical, natural disaster and epidemic, military, and fighting, the living dead and burden of care frames’ and presented ‘more neutrally with the alternative mind‐body frame’ [2, p. 10]. Their work provides a description of the ways in which dementia is represented across a range of texts in the English‐speaking world in recent decades. Gerritson et al. [3] argue that people with dementia are variously subjectified and treated according to the ways in which they are perceived and portrayed by others. They considered the influence of explanatory models of dementia, including biomedical, bio‐psycho‐social, and spiritual conceptions, person‐centred approaches, stereotypes and discursive understandings, media portrayals, and views of people living with dementia about themselves. The purpose of their work was to consider the moral implications of the ways in which people with dementia are viewed.

Together, these works illustrate the complexity of work among people living with dementia and their carers. First, their results identify a wide range of representations of dementia and people living with dementia. One of the common themes in their findings is the influence of the biomedical model in defining people with dementia. However, the representation of dementia is not limited to this technical perspective. Second, the ways in which people living with dementia are represented have implications for the ways in which they are treated, both individually, and as a cohort. This may result in benefits, such as treatment‐seeking behaviours [3], or in harms, such as reducing a person to being perceived as ‘a damaged brain’ [3, p. 598]. Third, their work was constrained to consider only people living with dementia and did not consider how others who are engaged with them are also subjectified. They also did not consider how this might impact the ways in which this wider cohort might be perceived. This includes carers, family, clinicians, care staff and researchers, among others who might also be identified by their relationships to dementia and people living with dementia. Finally, no common, over‐arching framework was identified or proposed that might unify approaches to work being undertaken amongst people living with dementia and their carers. It also did not accommodate the context of wider social arrangements engaging different agents and their roles when interacting with people with dementia. While each of these other frameworks has merit, having been derived empirically, neither takes the next step to provide a coherent theoretical approach. Their work might acknowledge the presence of clinical and non‐clinical outcomes of importance but does not account for them.

The purpose of this brief paper is to propose a unifying framework for the reporting of research outcomes that is based on different, but defined understandings of the Common Good, following the theoretical work of Boltanski and Thévenot [1]. We assert that this theoretical approach will enable broad application to the various types of outcomes that emerge from within different disciplines. Further, it provides a robustness that extends the application to the range of agents and entities engaged with people living with dementia and their carers, that is, to family and friends, clinicians, researchers and organisations, among others.

Application of this framework within the context of dementia‐related work has already been reported. Oldenhof et al. [4] applied the framework to consider the justificatory work of managers in the Dutch health care sector who had responsibility for overseeing small‐scale group homes for people living with dementia. Their study identified the managers' work of justifying actions, and defending and forging compromises, in the course of their duties. While identifying the varied work that managers found necessary to engage in, the study observed that the work of justification was achieved through discursive and material outputs, such as leaflets and budgets. Also, in consequence of the work of the managers, the persons with dementia were variously subjectified as clients, friends and members of families, members of civil society and consumers.

A different approach to the framework developed by Boltanki and Thevenot [1] was adopted by Schneider et al. [5], who sought to understand the subjective reality of domiciliary staff for people with dementia who were living in their own homes. Their analysis identified the activities and objects of importance to carers' experiences. Their study provided a descriptive analysis of what was important to domiciliary workers but did not assign the objects of significance to the categories of the Common Good, as identified in the EoW framework. We argue that while providing a description of what was important to the workers has value, there is benefit to be gained by making explicit the assumptions of the Common Good that remain otherwise implicit.

## 2 Definitions of the Common Good

In this paper, we seek to focus on persons living with dementia, and their carers, as the entities of primary interest, rather than on managers, or other staff. Six different definitions of the Common Good are described. Each definition and the associated principles and concepts identify a World of Valuing, which is applied to inform the ways that a person with dementia is variously subjectified. Additionally, implications for evaluating specific ‘good’ outcomes are illustrated. The World of Industry is described first as it is within this World of Valuing that technical measures support the assertion of good clinical outcomes. The other five Worlds are presented as offering alternative good outcomes, though not subjected to the same rigours of scientific and technical judgement.

Published articles were purposively sampled to provide a preliminary ‘proof of concept’ that different appeals to justice, consistent with the six different definitions of the Common Good, support the identification of a broad range of just outcomes for persons living with dementia. The discussion does not assume that the authors of the sampled studies were aware of the EoW framework. Rather, it assumes that the reporting of outcomes may be evaluated consistently with the categories provided by the framework. Table 1 lists key features of the Worlds of Valuing when applied to work amongst people living with dementia, and their carers.

**Table 1:** Summary of the six Worlds of Valuing, and how they might guide evaluation of outcomes related to dementia research.

World of Valuing	Higher common principle: the valuing of…	How to test the validity of a claim of good	How might the person living with dementia be subjectified?	Other people and objects that might be found within this world
Civic	… the collective	Representation	Citizen Voter Member	Clubs and social groups Residents' committees Memberships
Domestic	… tradition and hierarchy	Upholding tradition	Elder Vulnerable person Family member	Parents, children and carers Traditions and ancestries The family home
Industrial	… technical measures and efficiency	Technical measures	Patients (with diagnosis) Expert on own condition Research participant	Clinicians and scientists Standardised tests and measures Guidelines and procedures
Inspiration	… the outpouring of inspiration	Outpouring of inspiration (defies testing)	Mad person Spiritually insightful An inspiration	Visions and delusions Ghosts Priests and spiritual people
Market	… competitiveness	Monetary value	Customer Consumer Cost to the economy	Providers Budgets and accounts Service agreements
Opinion	… celebrity status	Public opinion	Famous identity (formerly or currently) Spokesperson Media manager	Accolades Celebrities and notable people Social media posts

### 2.1 The World of Industry

Technical and scientific measures and assessments support the World of Industry [1, pp. 203–211]. This World is structured around the Higher Common Principle of efficiency in order ‘to respond usefully to needs’ [1, p. 204]. In this World, those who function well are greatly valued, and the professionals and specialists who hold technical knowledge and skills have especial status. Justice is served within this World when tests and measures prove that functionality and reliability have been promoted. A breakdown of systems and subsequent inefficiency is judged as contrary to the principles of this World. Research outcomes can claim to serve the Common Good, according to the World of Industry, when they are supported by technical data and validated by technical experts. Research outcomes that fail technical tests, or that reduce people to technical objects, do not satisfy the criteria of justice defined by the Industrial World.

The study by Scher et al. [6] illustrates clinical research outcomes that were evaluated consistently within the World of Industry. In their study, carers were asked to rate the relevance, usefulness and acceptability of an animation that addressed the issue of grief when a loved one received a dementia diagnosis. The value of the research outcomes was clearly established by statistical measures. The relevance of their intervention was validated with 94% support from participants [6, p. 368]. However, the measures to determine usefulness and acceptability fell short of the benchmark of 75% (with a 95% confidence interval of 59%–87%) [6, 375]. Further evidence to support the statistics was found in the qualitative analysis of textual responses. Thus, the research can be argued to have served the Common Good when the matter of relevance was considered, but in a limited way for the qualities of usefulness and acceptability.

Clinical interventions may be tested and found to be beneficial but may also be evaluated as producing adverse outcomes. These may be signified by the technical measures, but also by reducing the human subject to a ‘technical object’. A negative outcome, ‘being researched on like guinea pigs’ [7, p. 574], was identified by the work of Mann and Hung [7], who described their co‐research between a lay researcher living with dementia (Jim Mann) and an academic researcher (Lillian Hung). Being subjectified as a ‘guinea pig’, while consistent with the World of Industry, signifies an adverse outcome in which instrumentalisation results in a denial of the dignity of human persons.

Thus, the Worlds of Valuing provide a framework for identifying benefits and harms arising from interventions. The following five Worlds describe how non‐clinical research outcomes might be evaluated from within their particular definitions of the Common Good. In this way, the technical valuing of the World of Industry may be placed in parallel with other systems of logic, thereby extending the observation of outcomes to include non‐clinical outcomes of value.

### 2.2 The World of Civics

The World of Civics [1, pp. 185–193] defines the Common Good according to the Higher Common Principle that places value on the collective. In this World, individuals are collected into organisational structures, which give them worth. Those who rule within the collective, or act as its representatives, hold elevated status within this World. Appeals to justice are consistent with conceptions of unity and solidarity, and are antithetical to isolation and individualism. Research among people living with dementia and their carers may be evaluated as good and just when it supports the collectivities to which people living with dementia, and their carers, belong. An outcome may be declared to serve the Common Good when it encourages participation in organisations, strengthens policies and procedures, acknowledges official offices, meetings and democratic processes. An outcome that does not serve the Common Good is one that disenfranchises people, isolates them, or erodes trust in the collective.

Participation in groups that promoted empowerment for people with dementia was shown to promote identity and the development of new opportunities for social participation and skills development in the investigation into the social integration of people with early‐stage dementia by Hagan and Campbell [8, p. 2366]. The group identity supported engagement with political processes influencing policy outcomes in Northern Ireland and was protective against isolation. This study serves as an example of good, non‐clinical outcomes consistent with the Civic World's description of the Common Good.

Grubb and Frederiksen [9] highlighted tension when volunteers sought to represent vulnerable persons, including persons living with dementia, and their carers, to municipal authorities. Their analysis identified policy, organisational structures, and authorised representatives engaged in a dispute to expand a programme. The dispute they identified was between the assertion that the expanded programme would serve the needs of the vulnerable citizens with dementia, and the assertion that the proposed programme was not truly representing public needs but was ‘entangled’ with ‘organisational positioning’ on the part of the volunteers. In this case, agreement about how the Common Good might be served was not achieved, and the negative evaluation of the programme resulted in its discontinuation.

### 2.3 The Domestic World

The Domestic World [1, pp. 164–178] does not restrict itself to household considerations. Its central interest is in relationships that reflect ‘engagement according to tradition’ [1, p. 165]. Those who hold ranks and titles, who are embedded in hierarchical structures, and who have connections are of especial worthiness according to this World. Appeals to respect and honour, in contrast to shame and dishonour, are consistent with the Common Good, as defined within this World. For a research outcome to be evaluated as good within this World, it must contribute to customs and conventions and uphold the lines of traditional authority. Outcomes that are deemed to challenge tradition, or that might be deemed impolite or embarrassing will be judged as not serving the Common Good.

While writing about ‘clients’, Oldenhof et al. [4] clearly identified people living with dementia as ‘family members’. As such, other relatives were invited to paint or decorate a client's room. Acknowledging a person's home, personalising it, and encouraging family involvement are evaluated as positive, though non‐clinical, outcomes aligned with the Domestic World. Vetter et al. [10] moved beyond clinical language to recognise ‘caregiver preparedness to care for their family members’ [10, p. 1246] thus validating a wider range of social definitions for their study cohort (family members and people living with dementia).

Complexity in the Domestic World was described in the work of Zhang et al. [11] amongst Chinese families. People living with dementia and their family carers were interviewed to understand their perspectives of family‐based care. The research identified that for some families, home‐based care strengthened a sense of family. This is regarded as a positive outcome from within the context of Domestic valuing. However, in other families, both the person living with dementia, and the family carers, reported that the person living with dementia was ‘useless’ and that death was a good alternative [11, p. 2829]. These responses are evaluated within the Domestic World as a failure to value elders. Thus, research findings may be evaluated as positive or negative, according to the logic that is defined within a specific World of Valuing.

### 2.4 The World of Inspiration

The World of Inspiration [1, pp. 159–164] contrasts with the World of Industry because it values what cannot be controlled because of the outpouring of inspiration. In this World, those who are in tune with the spiritual and imaginative are valued, along with the fantastical and phantasmal. Visionaries and eccentrics are applauded, whereas those who are grounded, rigid and boring are of little value. Research outcomes that serve the Common Good consistent with this World support creativity, lived experiences, and questing. Research outcomes that deny the ineffable and intuitive are not justified as serving the Common Good from within the World of Inspiration.

The quotation from a research participant that was used in the title of the report written by Hutmacher and Schouwink [12] suggests an exploration of the Common Good, according to the World of Inspiration: ‘it is the beautiful things that let us live’ [12, p. 403]. The creative activities that spouses, relatives and friends of people living with dementia engaged in varied between research participants and included playing music, making cards, keeping a journal or diary, walking in the forest or making a film documentary. This research gave rise to outcomes consistent with the World of Inspiration insomuch as it gave value to the lived experience of people engaging in creative and imaginative activities leading to personal discoveries and helping them to ‘embrace their destiny’ [12, p. 403]. By this assessment, research outcomes that engage the creative spirit may be justified as consistent with the Inspired description of the Common Good.

### 2.5 The Market World

The Market World [1, pp. 193–203] is ordered around the Higher Common Principle of competition, which is not focused on economics, per se, but on satisfying desires to acquire and possess. Those who possess rare and desirable commodities are regarded as being of high worth, whereas those who fail, or lose are of little worth. Justice is achieved, according to this World, when monetary value supports the claim that a good deal has been done. A lack of emotional detachment when engaging in the market is a necessary feature of this World, as it is protective against being possessed by the possession. Research outcomes that result in the acquisition of desirable goods may be judged as good according to the Market World, whereas outcomes that result in a loss of value do not satisfy the criteria of justice.

The systematic review of ‘the economic cost of dementia’ reported by Cantarero‐Prieto et al. [13] found differences in the health‐care costs for people with dementia varied between the EU and the USA. The severity of dementia meant a greater cost burden, according to their findings, thus defining aspects of dementia by monetary costs. They concluded that more work on economic matters in relation to dementia ‘would provide information to allow a [sic] better decision‐making about public‐health priorities in dementia’ [13, p. 2653].

Consideration of Market‐oriented thinking was demonstrated in the cost–benefit analysis of a programme offered to Australian veterans with dementia [14], for which dollar values were estimated for overall programme delivery, for individual sessions, and for individual participants per session. Together, the work of Cantarero‐Prieto, and of Meyer et al. [14] serve the Common Good, as defined within the Market World, where a monetary value was able to be assigned to different aspects of dementia, and associated interventions.

### 2.6 The World of Opinion

The World of Opinion, or Fame [1, pp. 178–185] may also be referred to as the World of Celebrity. Its central principle is the reality of public opinion, which evaluates the Common Good according to current, and changing public sentiment. Those who maintain high public visibility, public adoration and reputation have high value within this World, whereas those who are unknown or forgotten are of little worth. Research outcomes that are sensational, fashionable or backed by a high‐profile ‘celebrity’ are consistent with the definition of good within this World. Outcomes that do not capture the public imagination, or that remain relatively unknown do not serve the Common Good defined within this World.

Johnstone [15] critically examined the ways in which Alzheimer's disease was applied to influence the euthanasia debate. This study illustrates elements of the World of Opinion in two ways. First, the opinions of someone with a high public profile, Baroness Mary Warnock, were employed to influence the outcome of the debate as she sought to promote euthanasia in response to the suffering resulting from Alzheimer's disease. According to the World of Opinion, Baroness Mary Warnock was a person of high status, thus influencing what might be regarded as a good outcome in the debate. Second, the study sought to understand how public opinion was influenced by specific components of the debate of Alzheimer's disease and euthanasia. For example, it identified ‘morally loaded language’ associated with euthanasia that suggests beneficence to the public [15, p. 388]. The paper challenges the good that proponents of euthanasia implied when using their morally loaded language, or that was explicit in the public opinions of high‐profile persons.

## 3 Justification, Fairness, Violence and Love

**Figure 1:**
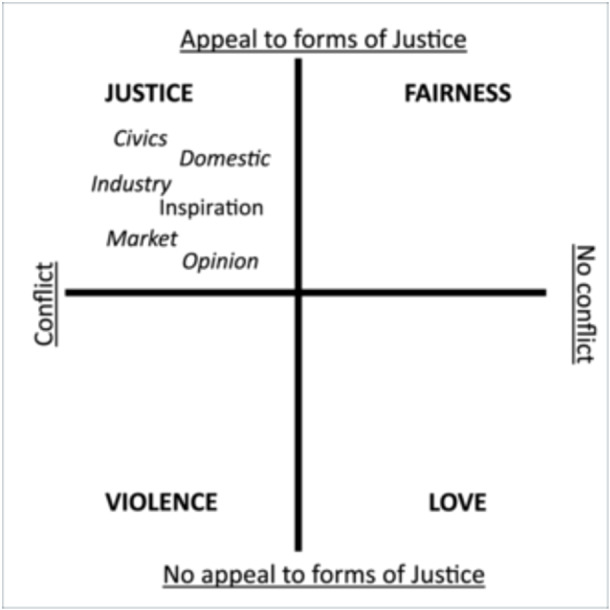
Situating the Worlds of Valuing within the Fields of action. *Source:* Adapted from Albertsen and Diken [16].

The act of justification is described by Boltanski and Thévenot as one possible action from a field of four actions that also includes acts of Fairness, Violence and Love (Figure 1, adapted from Albertsen and Diken [16]). Justification is defined as appeal to a form of the Common Good within the context of a dispute. Fairness is an action that involves appeal to the Common Good, but in the absence of dispute, such as when the necessity of washing the bodies of people with dementia is presented as an obvious action, though it still needs to be undertaken respectfully [17, p. 191]. Acts of Violence do not necessarily involve physical action. They are actions, in the context of a dispute, that seek to evade justice by shutting down discourse and appeals to any form of the Common Good ([1, pp. 37–38]; [18, pp. 72–73]). A review of articles studying abuse of older persons with dementia identified a range of abuse subtypes including psychological abuse, physical abuse and neglect [19]. The varied usage of the terms ‘violence’ and ‘abuse’ was also noted in the research. However, under the framework proposed here, the actions identified as abuse by Fang and Yan [19] would be included under the broad category of Violence. Finally, acts of Love are defined as those acts that do not require justification, but simply seek to accomplish good. A simple example is the giving of a gift without even the suggestion of needing praise or gratitude in response.

## 4 Future Research and Validation

This framework requires validation by scoping the various outcomes of clinical and non‐clinical research outcomes across a range of disciplines focused on providing interventions to address human need. Within the specific context of addressing dementia, identification of outcomes should evaluate whether the outcomes may be regarded as positive and beneficial, or as negative and harmful. As stated earlier, outcomes are attributed to a variety of entities related to the research, for example: by people living with dementia, by carers and families, by the researchers, or by community groups and organisations. A scoping review should therefore also identify who benefits (or is harmed) by the outcomes. From this review, a matrix may be developed that identifies the range of outcomes that are regarded as important in the reported literature. It should also identify the assumptions of the Common Good that underlie the reporting of the outcomes, the entities to whom the outcomes are attributed, and an evaluation of whether the outcomes are considered positive or negative.

Validation will be achieved when such a matrix is embedded as a guiding tool in research projects. The matrix would guide researchers when identifying the range of outcomes that might be intended. The practical adequacy of this framework will also be evident when applied to identify gaps in the delivery of interventions that address clinical and non‐clinical outcomes.

Finally, the framework, outlined here, may also be expanded to include other Worlds of Valuing that were described subsequent to the initial work of Boltanski and Thévenot. These include the Project‐Oriented World (or Network World) [20] oriented around the principle of flexibility, the Green World [21] centred on the principle of Sustainability, and the World of Diversity [22] focused on the principle of Inclusion. Including these other Worlds has the potential to expand the range of the good outcomes (or harms) that might be achieved and identified for clinical and non‐clinical research and their outcomes. Further, they broaden the ways in which those engaged with research may be understood, thus extending the range of social roles that they occupy. As for example, persons with dementia were described, not just as research subjects, but as parents, citizens and creative spirits.

Thus, the benefit of this pluralistic approach to defining persons engaged with research works against a reductive view of what constitutes them as persons. Simultaneously, it identifies the various types of Good that may be achieved when evaluating interventions and consequent outcomes arising from clinical and non‐clinical contexts. The application of a holistic framework such as this has the potential to unlock benefits (and harms) associated with research and community projects that may otherwise go unnoticed, unvalued, and even dismissed.

## Conflicts of Interest

The authors declare no conflicts of interest.

## Data Availability

The authors have nothing to report.
